# Development of the therapeutic language coding system (SICOLENTE): Reliability and construct validity

**DOI:** 10.1371/journal.pone.0209751

**Published:** 2018-12-26

**Authors:** Alberto Rodríguez-Morejón, Alberto Zamanillo, Gabriel Iglesias, Alberto Moreno-Gámez, Desirée Navas-Campaña, Patricia Moreno-Peral, José Luis Rodríguez-Arias

**Affiliations:** 1 Department of Personality, Assessment and Psychological Treatment, University of Malaga, Málaga, Spain; 2 Institute of Biomedical Research in Málaga, Málaga, Spain; 3 Prevention and Health Promotion Research Network, Málaga, Spain; 4 Independent Researcher, Málaga, Spain; 5 University Loyola Andalucia, Sevilla, Spain; 6 Research Unit, Primary Care District of Málaga-Guadalhorce, Málaga, Spain; 7 Complejo Hospitalario Universitario a Coruña (CHUAC), La Coruña, Spain; National University of Singapore, SINGAPORE

## Abstract

Since the use of language is a core aspect of psychotherapy, its study requires instruments that allow for further research. The aim of this study is to present an observational instrument capable of analyzing the language used in psychotherapeutic settings, both by therapists and clients. The SICOLENTE instrument was applied to two different samples: The *Three Approaches to Psychotherapy* film and a naturalistic sample. 7710 utterances from 31 sessions (three from the demonstration film and 28 from a naturalistic setting) were coded. Two studies were conducted: in the first study, inter and intra coder reliability (dimension and category levels) and Generalizability theory analyzes were assessed, whilst in the second study, construct validity was tested with several hypotheses. The final instrument resulted in 20 categories with three dimensions: Conversational Act (7 categories), Therapeutic topic (6 categories) and Content (7 categories). The three dimensions showed excellent inter and intra coder reliability and the generalizability coefficients were excellent. Out of the 24 validity hypothesis proposed,19 were accepted. The finding suggests that the SICOLENTE is a reliable and valid instrument that can be applied to investigate the performance of various theoretical models. Its three dimensional structure gives it the flexibility to be able to carry out macroscopic or microscopic language research.

## Introduction

Psychotherapy is a spoken profession and the conversation between the professional and the client is the central aspect of this work [1,2]. The different psychotherapeutic models have emphasized this dialogue in a range of ways. Since Freud [3] declared that "Words were originally magic, and to this day words have retained much of their ancient magical power" (p.17), many attempts have been made to develop that *magic* to make psychological treatments successful. Among the humanists, Rogers raised the importance of the therapist's expressions in building the therapeutic relationship [4]; whilst a Gestalt perspective teaches users that the responsibility they have for their lives begins in language [5]. Beck and Ellis’s influential cognitive therapies [6,7] focus their therapeutic potential on discussing client beliefs in sessions. Third-generation therapies focus on the direct or metaphorical use of language to make it easier for people to become detached from their thoughts [8]. However, it is the constructivist therapies that have assigned more importance to linguistic interaction as a tool for constructing changes in-session [9–12].

If language is the basic tool to produce changes in the session, analyzing its use is undoubtedly a relevant issue for research into the process of psychotherapy. The study of this process will help to optimize therapeutic communication with the consequent benefits of improving the therapeutic outcomes and the training programs for new therapists.

The analysis of language in psychotherapy generally requires the use of an observational system or methodology. Gumz, Treese, Marx, Strauss and Wendt [13] have conducted a systematic review that sheds light on the instruments created to date. In this review, the authors found 34 language analysis instruments that met the review eligibility criteria. Among the inclusion criteria, the most basic was to present data regarding the psychometric properties, and this review highlighted the limitations of the selected instruments. The main flaw is that only two of the instruments had been developed to measure the therapist-client interaction [14,15], while the rest of the instruments focused on measuring aspects such as adherence to treatment and competence [16–19], the difference between models [20–23] or the relationship between specific techniques and therapeutic outcomes [24–27]. In addition, the review found that all the instruments evaluated had a specific theoretical orientation—or several in the case of pantheoretical instruments, and there were no instruments that could be regarded as truly atheoretical.

The objective of this work is to present a new observational system of analysis, the System of Codification of Therapeutic Language–Sistema de Codificación del Lenguaje Terapéutico in Spanish or SICOLENTE, designed with the objective of overcoming both limitations. This system is concerned with studying the psychotherapist-client interaction. In addition, since the ability to use language for different purposes (create a therapeutic relationship, change meanings, help regulate emotions, or encourage new behaviors) is a cross-cutting and basic therapeutic element common to most therapeutics models, the instrument is developed without any bias towards specific therapeutic theories.

Previous research reveals that the language used by the therapist is influenced by the theoretical models with which they work [28–30]. In particular, evidence has been found to suggest that the therapeutic models condition the discussion topic of the clients during the session, allowing them to focus more on describing the problem or the improvements [31,32]. In this regard, the study by Cuhna et al., [33] showed how a certain therapist's language skills made it easier for the client to express moments of improvement during the session. Therapists also use those language skills to respond to issues expressed by the clients. In this way, there is a stronger likelihood that therapists will show responses of approval when clients verbalize aspects that favor the achievement of treatment goals (e.g., show greater well-being or verbalization of achievements) or responses of disapproval when the clients verbalize aspects that distance them from the therapeutic objectives (e.g., when the clients verbalize that they did not perform the task or anticipate problems regarding the treatment) [34,35].

### Hypothesis and work plan

The objective of this study is to present the SICOLENTE and describe its psychometric characteristics. For this, two studies were carried out. Given that this is an observational instrument aimed at analyzing the psychotherapist-client dialogue, the fundamental psychometric characteristics to be investigated are the instrument’s reliability (Study 1) and its construct validity (Study 2) [36]. For the first study, the aim was to evaluate the inter-coder and intra-coder reliability using Cohen's Kappa [37] and to carry out a study based on the Theory of Generalizability (G-theory) [38]. To assess construct validity, two experts hypothesized about the language of the therapists based on five different models (see Results section of Study 2).

### SICOLENTE: Creation of the instrument

The coding manual can be obtained on the web page https://osf.io/dyuz2/.

The observational instrument SICOLENTE was developed to fulfill the following four requirements: (a) to analyze all the verbal language present in a psychotherapy session, not only measuring the impact of a specific technique or a specific variable; (b) to investigate the language of the therapist, the client and the interaction between them; (c) to be able to study therapists of any model, i.e. to not respond only to the theoretical assumptions of a particular model, but instead work towards a way of understanding communication in therapy that will be discussed later; and (d) enable external observers to apply this model to study the participants of the therapist-client interaction.

For the design of the instrument, we worked on combining up-down and bottom-up strategies. The up-down strategy is based on a simple model that understands psychotherapy as an interactional process between a therapist and a client [39]. The therapist is formed in a model that adopts a series of theoretical assumptions: a way of understanding the human being, a theory that explains why people have problems, and a vision about what should be done so that problems can be solved. These theoretical assumptions are translated into practice in a series of procedures: two basic types of skills (creating a therapeutic relationship and promoting changes in meanings) and a varied repertoire of techniques. The theoretical assumptions vary substantially between models and each approach has its own set of techniques. Moreover, the basic linguistic abilities needed to create a relationship or work with meanings can be very similar between approaches, and this is the starting point of the SICOLENTE. From this perspective, therapy is a conversation for change in which the clients are the protagonists: they are the expert in their problem and have their own theories about why it exists and how it can be resolved [40]. Therefore, the ability of the therapist to adjust the treatments to people is understood as being key to the process and one of the aspects that should be measured by the SICOLENTE through language analysis. To implement the bottom-up strategy, we worked from an inductive approach in two phases. Firstly, a group of four expert clinicians made non-systematic observations of sessions of five different psychotherapy models to have an overview of the therapeutic process. The second phase began by recording a session in which two experienced therapists (a cognitive-behavioral and a systemic therapist) created an initial treatment session with a simulated client. The sessions were analyzed by twenty people with three distinct training levels: eight psychology students without knowledge of psychotherapy, seven doctoral students in psychology, and five clinical psychologists of different therapeutic orientations. Everyone is asked two questions: what therapeutic elements can be identified in the session? And what does the therapist do to try to bring about changes in the client? From their answers, the first ideas emerged for building the system.

From there, a team of six professionals (ARM, AMG, GI, DNC, PMP, and an external codifier) began to propose dimensions and categories, and through an iterative process of evaluating psychotherapy sessions, they obtained the final structure. In addition, a coding manual was developed with category examples and counterexamples, as well as the coding process.

### SICOLENTE: Instrument categories

The "language" construct that measures the final instrument consists of three dimensions (a) Conversational Act, (b) Therapeutic Topic and (c) Content, which correspond to the three classic dimensions of semiotics: pragmatics, semantics, and syntax respectively.

Each dimension has several categories, which are mutually exclusive and exhaustive (See Table 1). The first dimension, Conversational Act, asks "what are the psychotherapist and the client doing?" This dimension encompasses the aspect of language pragmatics and is the only dimension with different codes for psychotherapist and client. The codes for the therapist distinguish between current meaning, what they already share (e.g., the client's demographic information, their background, what they have talked about so far) and new meaning (e.g., asking for exceptions, reframing, interpreting, cognitive restructuring, and empty chairs). In addition, they distinguish themselves in terms of whether the therapist asks a question or if it is a statement. For the client, the codes are whether he or she follows or rejects the new or current meaning or changes the subject.

**Table 1:** Categories of the therapeutic language coding system (SICOLENTE).

**Conversational Act dimension: therapist**	
Exploration (E)	Any question from the therapist that seeks information but does not bring any new meaning to the conversation
Support (S)	They are repetitions, summaries, reflections, and everything that implies that the therapist follows the conversation and returns what he / she understands
New information (N)	Comments through which the therapist introduces information that was not present in the conversation, changes meanings or provides new relations among pieces of information
Exploration introducing new information (I)	Questions the therapist uses to add new information to the conversation, they may be used to change meanings or suggest new relations among pieces of information
Comment (C)	They do not fulfill the function of creating an alliance or introducing new visions, they are usually extra-therapeutic, or they are destined to formal questions (for example, schedule of the appointments)
**Conversational Act dimension: client**	
Follow (F)	The user seems to agree with the intervention of the therapist and continues with the conversation (at least there is no disagreement expressed)
Reject (R)	The user discusses to some degree the intervention of the therapist. It can be a clear disagreement, a clarification (for example: “yes, but”) or a sudden change of subject
**Therapeutic Topic dimension**	
Improvement (I)	Conversations which deal with improvements related to the problem consulted, or any positive aspect of people’s life and relationships (whether or not they are related to the complaint), or any aspect that is positively valued by the user and / or therapist
Problem (P)	Any conversation about what does not work and has been the reason for consultation, about any negative aspect of people’s lives (whether or not directly related to the client´s problem), or any conversation about something that is negatively valued by the user and / or the therapist
Goal (G)	Interventions about the objectives of the therapy or about the changes sought in people’s lives
Rules (R)	Conversations about the work rules that the therapist establishes or about aspects related to the therapeutic process (for example, appointments or length of the session)
Neutral (N)	The topic of conversation does not fit into any of the previous categories. They are usually descriptions of circumstances or situations that have not been valued positively or negatively and that are not about goals either
Mixed (X)	When an intervention talks about more than one therapeutic topic
**Content dimension**	
Behavior (B)	Conversations about the users’ behaviors; they are usually translated into action verbs
Thought (T)	It refers to users' cognitions, mental states, qualities, thought mechanisms, obsessions, etc
Emotion (E)	Conversations about sensations or feelings experienced by users
Physiology (P)	Refers to purely physical sensations, usually physiological symptoms that people perceive or physical states that experience such as tachycardia, sweating, sleep, trembling, etc.
Relationship (R)	When the content of the conversation involves more people and the client´s relationship with them
Mixed (X)	Interventions in which there are two or more ideas, with different specific contents, are categorized as mixed
Unspecific (U)	This category is used to encode contents that are either not very specific, or do not fit into any of the criteria of the previous codes

The second dimension, Therapeutic Topic, asks "What are they talking about?" This evaluates the topic and locates it in time (e.g., a good topic in the future is a goal, whilst a problem can be in the past, present, or future). This dimension is included within semantics i.e. the meaning (to the participants) of what is expressed in the dialogue.

Finally, the Content dimension asks "What action or user status is being referred to in the language?" It includes whether it is observable or not, intentional or not, and whether it was with or about another person. Given the fact that answering this dimension required the coding process to be centered on the verb used, this dimension is included in the syntax.

### Use of the instrument

The SICOLENTE is used with psychotherapy samples recorded in audio or video, in which only the verbal aspect is coded. The unit of analysis is a speech turn: the client’s utterance until the therapist speaks again and the intervention of the professional until the client speaks again.

Each intervention receives a unique code of three letters, a code for each dimension of the instrument. New three-letter codes are assigned as many times as there are changes in first dimension categories. Thus, the coding process always starts with the first dimension, and this generates the division in the data continuum.

To demonstrate how the instrument is used, an excerpt from Roger (R) 's demonstration with Gloria (G) will be used [41]:

1. R: […] I'd be glad to know whatever concerns you.
  Codification: Exploration; Problem; Thought
2. G: Well, right now I'm nervous
  Codification: Follow; Problem; Emotion
3. R: Mhm
  Codification: Support; Neutral; Unspecific
4. G: But I feel more comfortable the way you are talking in a low voice and I do not feel like you will be so harsh on me. But, ah …
  Codification: Follow; Improvement; Emotion
5. R: I hear the tremor in your voice, so I know you are …
  Codification: New information; Problem; Physiology

On turn (1), Roger's performance is encoded as Exploration in the first dimension since the therapist looks for client information. In the second dimension (1) this is codified as a Problem when referring to the action of what concerns you, i.e. what bothers the client. Finally, Thought is encoded in the third dimension since this is content over which control can be exercised but is not observable (in this case a concern). Gloria's turn (2) begins with Follow in the first dimension as she responds and follows the conversation. In the second dimension (2) this is coded as Problem since it refers to the client’s nerves when responding to " […] whatever concerns you". Finally, the third dimension is encoded as Emotion to be a sensation. In relation to turn (3), this is a back-channel, and is encoded as Support, since it does not provide any information, Neutral since it does not imply any aspect of the relevant therapy, and Unspecific because it does not refer to any state or action by Gloria. In turn (4), Gloria continues speaking so in the first dimension this is encoded as Follow, and in the second dimension this is encoded as Improvement since Gloria uses an adversary with respect to what she said previously and, in addition, the situation she expresses is positive (feeling comfortable). In the third dimension, this is still coded as Emotion since she is comfortable with how Roger speaks, generating a subordinate phrase so that only the content is understood.

Finally, in (5) this is coded as New information because Roger adds meaning to her nerves by talking about the tremor and relates it to the former. In the second dimension, Problem is coded as it refers back to a negative aspect of Gloria's life. Finally, in the third dimension, it is coded as Physiology since the action or state of the client to which Roger refers is the tremor, an observable but uncontrollable action (such as crying or tachycardia).

## Method

The Ethical review board (University of Malaga) approved the study and all ethical standards were followed in both studies (CEUMA: 14-2016-H).

### Study 1: Reliability

#### Participants

To investigate reliability, we analyzed three complete sessions from two therapists. The first therapist is a 60-year-old male who works using a brief systemic therapy (BST) model that he himself manualized [42] which includes solution-focused therapy (SFT) and the MRI strategic therapy (ST). He has more than 30 years of experience, and is a trainer supported by the main professional associations in the country (Spain) with various publications on the model he uses [43]. He carries out its work in a public health setting. The other therapist is a 48-year-old male who applied the cognitive-behavioral model in his work [6,7]. He is a trainer at the University of Malaga and has more than 15 years of experience working as a therapist in a psychological care service of this university. These three sessions were chosen randomly from the naturalistic sample obtained to investigate the validity of the instrument that was used in the second study. Thus, the sample consists of a first session conducted by the systemic therapist and the first and second session of a treatment program given by the cognitive-behavioral therapist. Both clients were women, aged 26 and 28, and both had anxiety disorders. All participants (therapists and clients) took part voluntarily in the investigation after reading and signing the informed consent form.

#### Procedure

Following the methodology proposed by Anguera [44], the sample was transcribed, generating a total of 816 interventions codifiable by the instrument. To codify, two teams of three people were created. These six coders had previously studied the definitive manual of the instrument (ARM, DNC, PMP in one team and AMG, GI, and an external codifier in the other). In principle, each observer codifies the interventions separately and meets with the other two people on the team to agree on codes. The strategies that were followed to agree the final codes are [44]: (a) first, if two or three observers determine a code, it is accepted as a team code. If in the individual coding the three judges codify an intervention in a different way, two possibilities are opened, (b) revision of the manual, to choose the most accurate code of the three; and if the disagreement has still not been resolved (c) more conservative codes are selected, preferably non-specific or neutral codes. The inter-observer reliability test was carried out by comparing the final data of each of the two teams. To establish the intra-group agreement, the first team re-encoded approximately half of the conversational turns three weeks later (454), and the new data were compared with those obtained in the first coding by the same team.

To continue investigating the characteristics of the instrument, analyzes were conducted using the theory of Generalizability, which is used to determine to what extent the accuracy or reliability of a measurement allows for generalizing the observations made to the set of all the observations of the field [38,45], in this case, language in therapy. A generalizability study (G-study) was designed with two random crossed facets: Observers (O, two levels) and Categories (C, 20 levels). With the G-study, the effects of different sources of error are estimated and it is confirmed whether the highest percentage of variance is due to the observers or attributed to the categories of the instrument [46].

#### Results and Discussion

The analyses conducted were: (1) Cohen's kappa index to calculate the inter and intra coder agreement by category, and (2) G-study.

The results obtained using the Cohen's kappa index indicate a high degree of concordance in the three dimensions of the SICOLENTE for the inter-coder test (see Table 2). In accordance with Bakeman and Quera's proposal to take into account the number of codes used and the prevalence of codes among other influential factors in the Kappa statistic [47], the results obtained by the evaluators in the inter-coder agreement was 95% observer accuracy for Conversational Act and 90% observer accuracy for Therapeutic Topic and Content. Following the criterion of Landis and Koch [48] for using the Cohen's Kappa statistic, all the results obtained by the coding team could be considered *almost perfect*. Similarly, using the criterion proposed by Fleiss [49] the indices obtained would fall into the category of *excellent*. Regarding agreements by category, it was found that the lower agreement was obtained for the Improvement, Mixed (*k* = .77) and Behavior categories (*k* = .79) with the strongest agreements being for Follow (k = .99), Support (*k* = .95), New information (*k* = .91) and Physiology (k = 1). The data from the intra-coder agreement test again show a high degree of agreement in the three dimensions of the SICOLENTE (see Table 3). These data reflect the consistency of the encodings, as well as the efficiency of the coding training process.

**Table 2:** Inter-coder results. Code frequencies and Cohen’s Kappa agreement.

Group one	Group two								
Conversational Act	E	S	N	I	C	F	R	Total	individual *kappa*
Exploration (E)	127	1	0	11	0	0	0	139	.90
Support (S)	1	135	3	1	0	0	0	140	.95
New information (N)	0	6	84	0	2	0	0	92	.91
Exploration introducing new information (I)	8	0	0	43	0	0	0	51	.80
Comment (C)	2	0	4	0	21	0	0	27	.83
Follow (F)	0	0	0	0	0	348	2	350	.99
Reject (R)	0	0	0	0	0	4	13	17	.81
Total	138	142	91	55	23	352	15	816	
	*Cohen’s Kappa* = .925								
Therapeutic Topic	I	P	G	X	N	R	-	Total	individual *kappa*
Improvement (I)	53	3	2	2	6	0	-	66	.77
Problem (P)	1	179	1	6	17	0	-	204	.89
Goal (G)	0	0	138	1	7	1	-	147	.89
Mixed (X)	0	3	1	26	1	0	-	31	.77
Neutral (N)	14	3	13	1	304	2	-	337	.83
Rules (R)	0	0	1	0	2	28	-	31	.90
Total	68	188	156	36	337	31	-	816	
	*Cohen’s Kappa* = .852								
Content	B	T	E	P	R	X	U	Total	individual *kappa*
Behavior (B)	58	2	0	0	2	3	4	69	.79
Thought (T)	3	136	1	0	2	6	7	155	.83
Emotion (E)	0	1	35	0	1	2	0	39	.87
Physiology (P)	0	0	0	5	0	0	0	5	1
Relationship (R)	0	4	0	0	108	0	4	116	.91
Mixed (X)	4	7	2	0	0	67	0	80	.82
Unspecific (U)	10	10	3	0	4	2	323	352	.89
Total	75	160	41	5	117	80	338	816	
	*Cohen’s Kappa* = .862								

**Table 3:** Intra-coder results. Code frequencies and Cohen’s Kappa agreement.

Group two (first time)	Group two (second time)								
Conversational Act	E	S	N	I	C	F	R	Total	individual *kappa*
Exploration (E)	103	0	0	1	0	0	0	104	.96
Support (S)	0	67	3	0	0	0	0	70	.97
New information (N)	0	1	30	0	0	0	0	31	.93
Exploration introducing new information (I)	5	0	0	29	0	0	0	34	.90
Comment (C)	2	0	0	0	11	0	0	11	1
Follow (F)	0	0	0	0	0	195	0	195	1
Reject (R)	0	0	0	0	0	0	9	9	1
Total	108	68	33	30	11	195	9	454	
	*Cohen’s Kappa* = .970								
Therapeutic Topic	I	P	G	X	N	R	-	Total	individual *kappa*
Improvement (I)	31	1	0	0	2	0	-	34	.93
Problem (P)	0	158	0	2	4	1	-	165	.92
Goal (G)	0	0	34	0	0	0	-	34	.98
Mixed (X)	1	2	1	21	0	0	-	25	.87
Neutral (N)	0	6	0	0	177	0	-	183	.95
Rules (R)	0	0	0	21	0	0	-	13	.96
Total	32	167	35	23	183	14	-	454	
	*Cohen’s Kappa* = .936								
Content	B	T	E	P	R	X	U	Total	individual *kappa*
Behavior (B)	34	1	0	0	3	3	0	41	.86
Thought (T)	0	82	0	0	1	0	0	83	.95
Emotion (E)	0	0	22	0	0	1	2	25	.85
Physiology (P)	0	0	0	0	0	0	0	0	-
Relationship (R)	1	1	3	0	99	0	0	104	.93
Mixed (X)	2	0	0	0	0	43	0	45	.93
Unspecific (U)	0	4	1	0	3	0	148	156	.95
Total	37	88	26	0	106	47	150	454	
	*Cohen’s Kappa* = .926								

The first generalizability study was designed to check the behavior of trained observers in relation to the categories, through a two crossed facets design (Observers/Categories, O/C). Identification of the sources of variance indicates that most of the variability is associated with Categories (99.84%), being zero for the Observer facet and low for the residual facet (0.156%). The generalizability coefficients obtained were excellent (.00 and .00), confirming that the categories describe in a heterogeneous way (they are exhaustive and mutually excluding) the measured construct, in our case the language. By inverting the design (Categories/Observers; C/O), emphasis is again placed on inter-observer reliability. The generalizability coefficients are again excellent (.99 and .99) demonstrating high reliability among the observers (see Table 4).

**Table 4:** Variance analysis: Two crossed facets design.

Facets	SS	df	MS	Variance component	SE	%
Categories	453626,6	19	23875,084	11928,674	3684,006	99,852
Observers	0	1	0	-0,887	0,274	0
Categories x Observers	337	19	17,737	17,737	5,474	0,148

### Study 2: Validity

The study of validity began after obtaining the results of Study 1. Two different samples were used to conduct the construct validity study: The *Three Approaches to Psychotherapy* [41] (TAP henceforth) recording and a clinical sample obtained in naturalistic settings. The TAP sample (described below) was chosen for several reasons: it is a recording widely used in this research field [50–58], and is easily accessible to any researcher, which facilitates the understanding and replicability of the present study. Secondly, since the three interviews with the same client were conducted by the creators of each of the models, the client can be considered a constant and the observed differences in interaction are due to the specific differences introduced by each model.

The clinical sample of naturalistic settings (described below) was selected because it is more representative of the final object of study in which the instrument will be used, that is, to provide greater external validity.

#### Participants

The recording [41] presents a demonstration given by the therapists Carl Rogers, Fritz Perls, and Albert Ellis. This recording was made with the intention of obtaining a representative session of client-centered therapy (CCT) [4], Gestalt therapy [5] and emotional-rational therapy [7] respectively. The three therapists interview the same person, Gloria, a 30-year-old woman who agreed to be recorded and interviewed. She comes to the consultation referring to problems related to men, as well as difficulties in adapting to divorce and taking care of her young daughter.

The therapists that comprise the second sample are those that have already been described in the Participants section of the first study. The total sample obtained with the two therapists is 15 cases, of which only the first and last session of each were investigated. This produced a total of 28 investigated sessions; 16 sessions of the systemic therapist (four successful and four failed treatments) and 12 sessions of the cognitive-behavioral therapist (four successful and three failed treatments). In all cases, these are individual sessions and the participants took part voluntarily in the investigation after reading and signing the informed consent form at the beginning of treatment. The final sample consisted of 14 women and one man, with an age range of between 21 and 43 years, and an average age of 29 years. Of the sample, 40% had anxiety disorders, 40% had adaptive disorders and 20% had depressive disorders according to the DSM IV-TR diagnostic manual [59].

#### Procedure

The three TAP sessions produced 702 speech turns. The speech was encoded directly using the original video recordings, and transcripts were only used to support video encoding. The language was coded using LINCE software [60] configured with the SICOLENTE categories. AZ carried out the codification of the three TAP sessions. Before starting this procedure, AZ checked for encoding reliability by coding the intra-coder test sample consisting of 454 speech turns. The test is considered to be acceptable when obtaining a Cohen’s Kappa index of at least 0.95 for each dimension, since this is considered comparable to the gold standard [47]. The natural settings sample was coded by a group made up of DNC, PMP, and an external codifier trained in the use of the SICOLENTE. An external coder was used to avoid possible biases that could be systematically produced by the expert coders. These 28 sessions produced 7008 speech turns. The recordings of this sample were in audio and were transcribed and coded by hand.

Whilst the transcription and coding process was being carried out, the main author (ARM) and a second university professor, expert in psychological treatments, hypothesized about the expected results depending on the theoretical model of each therapist, after consulting the summary of each of the theoretical models in a well-known psychotherapy manual [61]. The hypotheses were constructed to describe the differences and similarities expected among the therapists in terms of the particular theoretical models they adopt.

#### Results and Discussion

Both the hypotheses proposed, and the results obtained can be observed in Table 5.

**Table 5:** Hypotheses and results for the proportion comparison between therapists.

			Roger		Ellis		
Hypothesis		Accepted	%	*f*	%	*f*	*Z*
H1	the proportion of Support codes is higher in Roger’s sample than Ellis’ sample	✓	73.8	127	24.1	14	-6.72**
H2	the proportion of Improvement codes is higher in Roger’s sample than Ellis’ sample	✘	2.9	5	0	0	-1,31
H3	the proportion of Emotion codes is higher in Roger’s sample than Ellis’ sample	✓	12.8	22	1.7	1	-2,44*
			**Perls**		**Ellis**		
			**%**	***f***	**%**	***f***	***Z***
H4	the proportion of Support codes is higher in Perls’ sample than Ellis’ sample	✘	17.0	23	24.1	14	1,15
H5	the proportion of Improvement codes is higher in Perls’ sample than Ellis’ sample	✘	3.7	5	0	0	-1,48
H6	the proportion of Emotion codes is higher in Perls’ sample than Ellis’ sample	✘	6.7	9	1.7	1	-1,43
			**Perls**		**Roger**		
			**%**	***f***	**%**	***f***	***Z***
H7	the proportion of Support codes is higher in Roger’s sample than Perls’ sample	✓	17.0	23	73.8	127	9.88**
H8	the proportion of New Meaning codes is higher in Perls’ sample than Roger’s sample	✓	44.4	60	19.8	34	-4,64**
H9	the proportion of Exploration INM codes is higher in Perls’ sample than Roger’s sample	✓	21.5	29	2.9	5	-5,15**
			**Ellis**		**Roger**		
			**%**	***f***	**%**	***f***	***Z***
H10	the proportion of New Meaning codes is higher in Ellis’ sample than Roger’s sample	✓	56.9	33	19.8	34	-5.38**
H11	the proportion of Exploration codes is higher in Ellis’ sample than Roger’s sample	✓	6.9	4	0.6	1	-2.84**
H12	the proportion of Problem codes is higher in Ellis’ sample than Roger’s sample	✓	51.7	30	30.8	53	-2.87**
H13	the proportion of Thought codes is higher in Ellis’ sample than Roger’s sample	✓	27.6	16	8.1	14	-3,82**
			**Ellis**		**Perls**		
			**%**	***f***	**%**	***f***	***Z***
H14	the proportion of New Meaning codes is higher in Ellis’ sample than Perls’ sample	✓	56.9	33	44.4	60	1,59
H15	the proportion of Thought codes is higher in Ellis’ sample than Perls’ sample	✓	27.6	16	5.9	8	-4,19**
			**CBT**		**SFT**		
			**%**	***f***	**%**	***f***	***Z***
H16	the proportion of Support codes is equal in CBT sample as the SFT sample	✓	31.1	466	31.9	701	0.51
H17	the proportion of New Meaning codes is higher in SFT sample than CBT sample	✓	20.1	301	33	725	8.60**
H18	the proportion of Exploration codes is higher in CBT sample than SFT sample	✓	36.9	553	19.4	427	-11.84**
H19	the proportion of Goals codes is higher in SFT sample than CBT sample	✓	6.3	95	12.3	270	6.01**
H20	the proportion of Improvements codes is higher in SFT sample than CBT sample	✓	7.9	118	16.2	357	7.41**
H21	the proportion of Problem codes is higher in CBT sample than SFT sample	✓	35.2	527	12.7	279	-16.26**
H22	the proportion of Thought codes is higher in CBT sample than SFT sample	✓	21	315	15.8	347	-4.05**
H23	the proportion of New Meaning codes is equal in SFT sample as the CBT sample	✓	15.4	231	13.7	302	-1.45
H24	the proportion of Relationship codes is higher in SFT sample than CBT sample	✘	13.3	199	14.1	311	0.69

✓ = the hypothesis is accepted

✘ = the hypothesis is rejected

CBT = cognitive-behavioral therapy; SFT = solution-focused therapy

* Z ≥ ± 1.96 = *p* < .05

** Z ≥ ± 2.58 = *p* < .01.

The analyzes conducted with the validity samples were: (a) comparison of proportions between pairs of therapists through Pearson chi-square (Roger-Ellis, Perls-Ellis, Roger-Perls, systemic therapist-cognitive behavioral therapist); for the test of comparison of specific proportions by codes, we chose to accept as significant the results in which the residual residuals were Z ≤ ± 2.58 = p < .01, and (b) sequential analysis of the therapist-client interaction. The proposed sequential analyzes study the conditional probability that a specific message of the speaker will be followed up by the addressee. For example, the therapist may be speaking about the client’s improvements (Improvement code) and the sequential analyzes calculate the proportion of times that the clients continue to talk about Improvement, or in what proportion they change the topic to Problems or Goals. Since the conditional probabilities in the sequential studies are expressed as percentages [47], comparisons of proportions were made, accepting as significant the results in which the adjusted residuals were Z ≤ ± 1.96 = *p* < .05. These analyzes were conducted to confirm the hypothesized results.

To compare the hypotheses proposed, the analysis begins by comparing the two humanistic therapists (Rogers and Perls) with the cognitive therapist (Ellis) from the TAP sample. The fundamental strategy in humanistic therapies is that people feel validated (Conversational Act: Support). Humanists share the idea that people are in a constant process of *self-realization* or *self-actualization* and, to overcome their problems, they must release their resources (Therapeutic Topic: Improvements), with emotional content being the core aspect of the treatment (Content: Emotion).

The analyzes confirm that Rogers showed significantly more Support codes than Ellis (H1: Z = -6.72, p < .01) and speaks more of emotions than the cognitive author (H3: Z = -2.44, *p* < .05). However, H2 cannot be accepted, since the Improvement code was not used more frequently by Rogers (H2: Z = -1.35).

In the case of Perls, none of the hypothesized differences were found: he does not show more Support than Ellis (H4: Z = 1.15), he does not talk about Improvement (H5: Z = -1.48), nor does he use emotional content more frequently (H6: Z = -1.43).

With respect to the theory, we expected to find differences in style between the two humanistic therapists. In particular, we anticipated that Rogers will focus on understanding and supporting, introducing very few new meanings, whilst the Gestalt therapist will introduce more new meanings in an attempt to favor the *awareness of here-and-now* (Conversational Act: Support, New information, Exploration introducing new information). The analyzes confirmed the proposed hypotheses: Rogers used the Support code significantly more (H7: Z = 9.88, *p* < .01) whilst Perls made more use of the New Information code (H8: Z = -4.64, *p* < .01) and that of Exploration and Introducing new information (H9: Z = -5.15, *p* < .01).

In comparison with the humanist approach, cognitive therapy has the clearer aim of helping people to set work objectives, using the initial sessions of problem evaluation to reeducate the client by challenging irrational cognitions and replacing them with more rational thoughts and beliefs (Conversational Act: Exploration, New information and Exploration Introducing new information; Therapeutic Topic: Problem; Content Thought).

In the sample studied, The New information code was significantly higher for Ellis than Rogers (H10: Z = 5.38, *p* < .01). Ellis asked Gloria more exploratory questions in comparison with Rogers (H11: Z = -2.84, *p* < .01) and guided the conversation towards a discussion of his client’s problems (H12: Z = -2.87, *p* < .01). With respect to the Content dimension, Ellis spoke significantly more of Gloria's beliefs and thoughts than Roger (H13: Z = -3.82, *p* < .01). With respect to the comparison of Ellis versus Perls, the results allow us to accept hypothesis H14, since there were no significant differences between the therapists in terms of the use of the New information code (Z = 1.59) and hypothesis H15 is also supported since Ellis speaks more about thoughts and cognitions than Perls (H15: Z = -4.19, *p* < .01).

In regards the naturalistic settings sample, both therapists are experts and work according to a model in which creating a good therapeutic relationship is essential to initiate therapy (Conversational Act: Support). Being a systemic solution-focused therapist, there is a clear commitment to changing meaning from the beginning of treatment (Conversational Act: New information or Exploration introducing new information). The aims of the solution-focused therapist in the first session are to establish objectives and analyze the exceptions, that is, what already works (Therapeutic Topic: Goals and Improvements). For the cognitive-behavioral therapist, the first session is eminently exploratory (Conversational Act: Exploration), with a focus on trying to understand the problem (Therapeutic Topic: Problem) to plan the subsequent treatment. The preferred work contents of cognitive-behavioral therapists will be thoughts and behavior, and it is expected that in the systemic therapist more relational codes will appear.

The analyses provide support for hypothesis H16, since there is no difference between the therapists in the use of Support codes (H16: Z = 0.51). As expected, the systemic therapist showed significantly more New information codes (H17: Z = 8.60, *p* < .01), while the cognitive therapist showed significantly more Exploration codes (H18: Z = -11.84, *p* < .01). Regarding the Therapeutic Topic, the results allow hypotheses H19 and H20 to be accepted; the systemic therapist’s proportions are significantly higher in the codes for Goals (Z = 6.01, *p* < .01) and Improvements (Z = 7.41, *p* < .01), whereas the cognitive therapist speaks more of Problems (H21: Z = -16.26, *p* < .01). In the Content dimension, the results allow hypothesis H22 to be accepted, since the cognitive therapist spoke significantly more about the clients' thoughts and beliefs (Z = -4.05, *p* < .01) and H23 can also be accepted because the therapists did not differ in terms of the use of the Behavior code (Z = -1.45). Finally, hypothesis H24 is rejected since no significant differences were found in the use of the Relationship code, which was expected to be higher in the case of the systemic therapist (Z = 0.69).

#### Sequential analyzes

In a system such as the SICOLENTE, which uses numerous codes and combinations, there are a multitude of possibilities for analysis and, therefore, the analyses carried out are guided by previous data. For instance, we already know that the systemic therapist proposes more conversations about positive aspects than the cognitive therapist (Improvement and Goals codes) and that the cognitive therapist proposes more conversations about the problems (Problem code). Thus, after all the hypotheses of the study were verified, the sequential analyzes were carried out, in which the conditional probabilities of occurrence were compared. In this case, the client-therapist interaction was investigated in the naturalistic sample, with a sequence of one prospective lag. The results show that the conditional probability of the therapist continuing to talk about improvements (Improvement code) after the client has discussed an improvement is .47 for the SF therapist and .35 for the CB therapist, this difference being significant (Z = 2.23, *p* < .05). Along with this, these analyzes allow us to know that when the client verbalizes an improvement, there is a conditional probability of .16 that the cognitive therapist continues talking about the problem as opposed to a low probability that the systemic therapist will do so (.03) (Z = -4.86, *p* < .01). Similarly, when the client's language is encoded in the Therapeutic Topic dimension as Mixed, (the client has expressed both positive and negative topics in the same turn), it is found that therapists choose the preferred topic of the theoretical model. Thus, the SF therapist tends to answer with a probability of .31 with the Improvement code, whilst this probability is .13 for the CB therapist (Z = 2.82, *p* < .01). The CB therapist tends to respond with the Problem code with a conditional probability of .35, which is .13 for the systemic therapist (Z = -3.38, *p* < .01).

## General Discussion

The main objective of this study was to present the SICOLENTE instrument and to check its psychometric characteristics. The final categories were nested in three dimensions: Conversational Act, Therapeutic Topic, and Content. Reliability and construct validity properties were investigated in two studies to warrant the use of this instrument in scientific research.

The calculated inter-coder reliability indices are excellent according to the criteria used, and this is true of each of the three dimensions of the instrument and its corresponding categories. Equivalent results were obtained when calculating intra-coder reliability. The conclusion is that well-trained observers, with a manual and a training program, obtain equivalent results and that these remain constant over time. It should also be noted that when working with a single coder instead of a team, the inter-coder score remained stable, which reduces the workload whilst maintaining the quality of the work. The analysis carried out on the basis of the G-Theory allows us to confirm that the variability of the obtained data is associated with the categories and not with other aspects (observers or residual); that is, the differences found are due to the instrument and not to the observers who applied it.

Regarding the construct validity of the instrument, this was verified by testing the ability of the SICOLENTE to differentiate therapists from five different models in terms of the proportion and code relationships, based on the evidence from previous studies showing that theoretical models influence the language used by the therapist [28,29,32]. The results have allowed us to accept most of the hypotheses presented. In particular, the SICOLENTE detects clear differences between the two modern therapists, and eight of the nine hypotheses are accepted (88.89% accepted). In the Conversational Act dimension there is no difference between them in the use of Support (S), which is the dimension upon which the therapeutic relationship is created. However, differences were found in terms the work strategy; the CB therapist showed more Exploration (E) whilst the SF therapist initiates considerably more interactions for changing meaning (N, I). In the Therapeutic Topic, both therapists behaved according to the model. In particular, the CB therapist focused more on problem conversations (P) and the SF therapist referred more often to Goals (G) and Improvements (I). In terms of the third dimension, the results for Content support one of the predicted hypotheses, that is, the CB therapist speaks more of Thoughts (T), although the SF therapist failed to show a higher proportion of use of the Relationship code (R). This last result could be due to the fact that the cases analyzed are all individual.

The results are less clear regarding the analysis of the three classical therapists. In spite of this, 11 of the 15 hypotheses are accepted (73.34% accepted). Rogers differs from Ellis in that he was more concerned with validating the client (S) while Ellis introduced more new meanings (N, I) and focused the conversation more on the problem. Perls, on the other hand, appeared to behave more like Ellis than Rogers, since he was more concerned with creating new meanings (N, I) rather than validating his client (S). These results are consistent with those of previous research, in which Perls' performance is characterized as challenging and confronting [52,54,62]. This discrepancy between model and practice can be explained by the small sample size used or by the performance-like context of the record, which encourages the therapists to demonstrate the efficiency of their model in a short space of time. None of the humanistic therapists appeared to be particularly interested in highlighting the positive aspects (Improvements) of their clients, as might be expected *a priori* when working with models based on the idea of self-realization; an aspect that appears to be central to the performance of the SF therapist. The results obtained, although not hypothesized, are in accord with those described by Tomori and Bavelas [32], who compared the language of two SFT therapists with that of two CCT therapists and found that while the SFT construct conversations were centered around positive aspects (Goals and Improvements) CCTs focused their conversation on the problem.

One question that arises when analyzing the results is: why do modern therapists seem to behave more in line with their models than classical therapists? To begin with, as already mentioned, the context of the TAP recordings [41] encourages personal recognition, and it detaches the performance of the therapist from the theoretical model. Moreover, in recent years, therapy has been manualized (one of the modern therapists is the author of a brief therapy manual in Spanish) so it is more likely that the therapists of the validity study will be more faithful to the directive of the model with which they are affiliated.

Finally, the sequential analyzes conducted demonstrate the usefulness of the SICOLENTE when it comes to understanding how therapists manage to maintain the conversational preferences determined by the model. Cognitive therapists tend to talk more about problems, but they also continue to talk about this topic when the client proposes the issue, which is also shown for systemics with the Improvement topic. It is also clear that therapists skew the conversation towards the preferred topic. In the case of mixed codes, the CB therapist is more likely to steer towards a problem while the SF therapist is more likely to lead the conversation towards Improvement.

### Conclusions

We can conclude by affirming that the SICOLENTE instrument is useful for describing and differentiating the in-session use of language by therapists, according to the model that they adopt in their work. Our unexpected findings can be attributed both to deficiencies in sensitivity of the instrument, which will require correction, and also the possibility that some of the therapists studied are not entirely faithful to the theory they endorse, perhaps because of the cinematographic context in which the studied recording was made.

### Limitations

Several limitations of this study are worthy of note: a) The SICOLENTE only analyzes the verbal aspects of the psychotherapist-client interaction, which prevents us from knowing the effect it has on the dialogue related to other communicational elements such as intonation, facial gestures, or hand gestures [63]; b) In this study only individual therapies have been coded and it would be advisable to think about the modifications that are necessary to codify sessions in which there is more than one client.

Another aspect of the present study that requires improvement is the size of the sample used. The Cohen's kappa statistic is influenced by coding systems in which there is a category of low frequency (the client’s category Reject, for example; [37]), which could generate an artifice in the other indices obtained, although the inter-coder agreement was very high in the less frequent categories. In any case, increasing the sample size could alleviate this problem. Similarly, the possibilities of the instrument are greater if we work with a combination of two or three dimensions, which would cause some of the code combinations to have even lower frequencies, which again leads to the need to work with larger linguistic samples.

### Future research

Thanks to the triple code system, macroscopic investigations can be conducted to study the dimensions separately, (e.g. proportion of Support codes versus proportion of New information codes; proportion of Behavior codes versus Thought codes, etc.) whilst microscopic research can also be conducted using a combination of two or three dimensions together. For example, researchers can investigate “how many questions (Exploration code) about the problem (Problem code) at an emotional level (Emotion code) are asked in sexual abuse treatment” versus how many questions are asked about the problem at the behavioral level (Behavior code)”. Another example could be: Are New information codes introduced more frequently when the therapist is talking about the client’s beliefs (Thought code) or when he/she is talking about client relationships? And, is this new meaning related to the client problem or the client goals? Furthermore, the therapist-client interaction could be examined in the way described here or with more complex procedures. For example, one could investigate the response tendency of the therapist after the client rejects a new meaning, or it is possible to examine the effect of back-channels when clients are talking about their goals.

Finally, this was the first attempt to assess the reliability and validity of this instrument and should therefore serve to help its improvement. On the basis of these preliminary data, it will be necessary to review the following:

- The categories related to validating (S) or introducing new meanings (N, I). Whilst these have been particular relevant when it comes to understanding the therapeutic language, it would be convenient to think of subtypes of each of these categories that allow us to make richer descriptions of a central process in psychotherapy, that is, the balance between validations and the creation of new meanings.
- The high percentages in Neutral (N), Unspecific (U) or Mixed (X) categories could be indicative of the need for greater refinement of the instrument. The neutral or nonspecific codes are attributed to rather ambiguous messages that do not fit into another category, which is information that is lost. In contrast, for the Mixed (X) categories, the case is quite the opposite; there is a lot of information, but of at least two different codes, which could perhaps be better exploited if subtypes were created in the category, both in Therapeutic Topic and in Content.

Regarding the validity of the instrument, it is necessary to continue investigating this aspect by conducting studies on criterion validity. An alternative for the future would be to compare the SICOLENTE with other similar instruments.
